# The Ms6 Mycolyl-Arabinogalactan Esterase LysB is Essential for an Efficient Mycobacteriophage-Induced Lysis

**DOI:** 10.3390/v9110343

**Published:** 2017-11-17

**Authors:** Adriano M. Gigante, Cheri M. Hampton, Rebecca S. Dillard, Filipa Gil, Maria João Catalão, José Moniz-Pereira, Elizabeth R. Wright, Madalena Pimentel

**Affiliations:** 1 Research Institute for Medicines (iMed.ULisboa), Faculdade de Farmácia, Universidade de Lisboa, Lisbon, 1649-003, Portugal; amsgigante@ff.ulisboa.pt (A.M.G.); filipagil@hotmail.com (F.G.); mjcatalao@ff.ulisboa.pt (M.J.C.); jpereira@ff.ulisboa.pt (J.M.-P.); 2 Division of Pediatric Infectious Diseases, Emory University School of Medicine, Children’s Healthcare of Atlanta, Atlanta, GA, 30345, USA; cheri.m.hampton@emory.edu (C.M.H.); rebecca.s.dillard@emory.edu (R.S.D.); erwrigh@emory.edu (E.R.W.)

**Keywords:** bacteriophage lysis, mycobacteriophage, Ms6, LysB, mycobacteria, spanins, cryo-electron microscopy

## Abstract

All dsDNA phages encode two proteins involved in host lysis, an endolysin and a holin that target the peptidoglycan and cytoplasmic membrane, respectively. Bacteriophages that infect Gram-negative bacteria encode additional proteins, the spanins, involved in disruption of the outer membrane. Recently, a gene located in the lytic cassette was identified in the genomes of mycobacteriophages, which encodes a protein (LysB) with mycolyl-arabinogalactan esterase activity. Taking in consideration the complex mycobacterial cell envelope that mycobacteriophages encounter during their life cycle, it is valuable to evaluate the role of these proteins in lysis. In the present work, we constructed an Ms6 mutant defective on *lysB* and showed that Ms6 LysB has an important role in lysis. In the absence of LysB, lysis still occurs but the newly synthesized phage particles are deficiently released to the environment. Using cryo-electron microscopy and tomography to register the changes in the lysis phenotype, we show that at 150 min post-adsorption, mycobacteria cells are incompletely lysed and phage particles are retained inside the cell, while cells infected with Ms6*wt* are completely lysed. Our results confirm that Ms6 LysB is necessary for an efficient lysis of *Mycobacterium smegmatis*, acting, similarly to spanins, in the third step of the lysis process.

## 1. Introduction

Bacteriophages, the viruses of bacteria, are key elements for biosphere equilibrium, playing a fundamental role in bacterial evolution through constant interactions with their hosts [1,2]. To guarantee their own survival, double-stranded DNA (dsDNA) phages, which represent more than 95% of known bacterial viruses [3], must lyse their hosts. At the end of a lytic cycle, the new phage particles need to be released into the environment, where new host bacteria are potentially available for new infection cycles. The main barrier to phage release is the bacterial cell envelope, and thus, compromising this barrier is the main goal of the lytic process. To accomplish this goal, dsDNA phages synthesize two essential lysis proteins, endolysins and holins. Endolysins are enzymes that disrupt the bacterial cell wall (CW) by cleaving one or more of the five bonds in peptidoglycan (PG). Holins are small proteins that accumulate in the cytoplasmic membrane (CM) and that, at a genetically defined time, form holes in this cell membrane allowing the access of active endolysins to the PG layer or the activation of previously exported endolysins [4,5]. Phages that infect Gram-positive hosts only require the synthesis of these two proteins to compromise the bacterial envelope and consequently for cell burst. However, phages that infect Gram-negative hosts have to face an additional barrier, the outer membrane (OM). It has been shown recently that disruption of this barrier is also required for cell lysis [6]. This is achieved by a third class of lysis proteins named spanins. The best studied spanins are the λ Rz and Rz1 proteins which are an inner membrane and outer membrane protein, respectively. These two proteins form a complex that spans the entire periplasm mediating the fusion of the CM with the OM. This results in the elimination of the last barrier to phage release and consequently, lysis of the host [7,8,9]. Spanin genes, which may encode a sole protein (T1 Gp11) or two subunits like the λ Rz and Rz1 proteins, have been identified in nearly all phages infecting Gram-negative hosts [10,11]. This indicates that, for phages infecting Gram negative hosts, lysis is a three-step event where each component of the cell envelope, i.e., CM, CW and OM is sequentially attacked by holins, endolysins and spanins, respectively [9].

**Figure 1:**
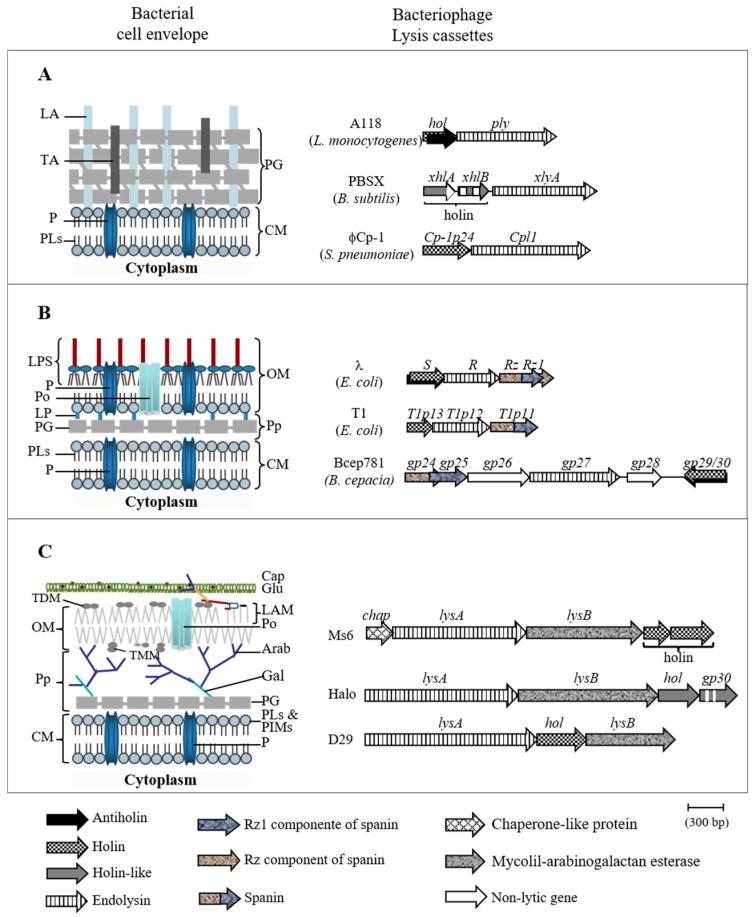
Cell envelopes of bacteria (left) and representative lysis cassettes of their infecting phages (right). (**A**) Gram-positive bacteria; (**B**) Gram-negative bacteria; (**C**) Mycobacteria. The white segments in holin-like genes indicate the number and position of transmembrane domain coding sequences. Abbreviations: CapGlu, capsular glucan CM, cytoplasmic membrane; LA, lypoteichoic acid; LAM, lipoarabinomannan; LP, lipoprotein; LPS, lipopolysaccharide; P, protein; PG, peptidoglycan; PIMs, phosphatidylinositol mannosides; PLs, phospholipids; PO, porin; Pp, periplasm; TDM, trehalose dimycolate; TMM, trehalose monomycolate. Adapted from reference [5] with permission.

Studies of mycobacteriophage Ms6, a phage that infects *Mycobacterium smegmatis*, have shown that the lysis cassette composition reflects the complexity of the cell envelope of its host [12]. Although mycobacteria are classified as Gram-positive bacteria, they have a complex cell envelope composed of a CM, similar to other bacterial CMs [13,14], surrounded by a peptidoglycan layer covalently linked to arabinogalactan (AG) which is in turn esterified to a mycolic acid (MA), forming the mycolyl arabinogalactan-peptidoglycan (mAGP) complex [15]. The MAs are long fatty acids that constitute the inner leaflet of a true OM. The outermost leaflet is composed of various glycolipids, including trehalose mono and dimycolate, phospholipids and species-specific lipids [16,17]. Finally, a capsule is composed of proteins, polysaccharides and a few lipids [18,19]. Thus, phages that infect mycobacteria have to overcome this complex envelope for a successful infective cycle. The Ms6 lysis cassette is composed of five genes [20] (Figure 1). In addition to the holin and the endolysin functions, Ms6 encodes a chaperone-like protein (Gp1) that is involved in the delivery of the endolysin to the PG [21,22,23] and an additional lysis protein, Lysin B (LysB), identified as a lipolytic enzyme with the ability to cleave ester bonds of both short and long fatty acids [24]. Experiments with components of the mycobacterial cell envelope showed that Ms6 LysB is a mycolyl-arabinogalactan esterase that cleaves the ester bond between the mycolic acids and the arabinogalactan, and this allows the separation of the OM from the CW [25]. Analogies can be made between Ms6 LysB and the spanins, where Ms6 LysB functions to mediate the final step of host cell lysis.

In the present work, we examine the importance of Ms6 LysB in phage lysis and taking advantage of cryo-electron microscopy (cryo-EM) and tomography (cryo-ET), we compare the Ms6 wild-type lysis phenotype with that of a Ms6 mutant lacking the *lysB* gene. We present evidence that absence of LysB in the Ms6 infection cycle results in incomplete lysis and suggest that the LysB role in lysis parallels that of spanins.

## 2. Materials and Methods

### 2.1. Bacterial Strains, Phages, Plasmids and Culture Conditions

Mycobacteria strains, phages, plasmids and oligonucleotides used in this study are listed in Table 1. *M. smegmatis* strains were propagated in 7H9 medium (BD Biosciences, San Jose, CA, USA) with shaking or Middlebrook 7H10 (BD Biosciences), supplemented with 0.5% glucose, at 37 °C. When appropriate, 1 mM CaCl_2_ or 15 µg/mL kanamycin was also added to the media. For induced conditions, cells were grown in 7H9 supplemented with 0.2% succinate and induced with 0.2% acetamide.

### 2.2. Construction of Ms6 Mutant Phage

Construction of Ms6 mutant phage was performed using Bacteriophage Recombineering of Electroporated DNA (BRED) in *M. smegmatis* as described previously [21,30]. Briefly, for deletion of gene *lysB* from the Ms6 genome*,* a 100 bp oligonucleotide (Pr∆*lysB)*, with 50 bp of homology to either flanking region to be deleted was generated. This fragment was extended by PCR to a 200 bp dsDNA substrate using two 75 bp extender primers, PrExt∆*lysB*Fw and PrExt∆*lysB*Rv, sharing 25 bp of homology with either end of the 100-mer. After purification, using MinElute PCR Purification Kit (QIAGEN, Hilden, Germany), the 200 bp substrate was co-electroporated with Ms6*wt* DNA into electrocompetent recombineering cells of *M. smegmatis* mc^2^155:pJV53. Cells were resuspended in 7H9 supplemented with glucose and CaCl_2_, incubated at 37 °C for 2 h (prior to lysis) and plated on top agar lawns with *M. smegmatis* mc^2^155. Individual plaques were recovered and eluted in 100 µL of phage buffer (10 mM Tris-HCl, pH 7.5, 10 mM MgSO_4_, 68.5 mM NaCl, 1 mM CaCl_2_), for two hours at room temperature and analyzed by Deletion Amplification Detection Assay (DADA)-PCR [30] with primers DADA ∆*lysB*-PCRFw/DADA Ms6-PCRRv to detect *lysB* deletion. Mixed primary plaques containing both wild-type and mutant alleles were eluted as described above, and serial dilutions were plated with *M. smegmatis*. Individual secondary plaques were screened by DADA-PCR using the same pair of primers.

### 2.3. Plasmid Construction

To construct plasmid pAG1, a DNA fragment containing the *lysB* gene was obtained by PCR amplification using Ms6 genomic DNA as template, with primers Pr*lysB*Fw/Pr*lysB*Rv and Pfu high-fidelity polymerase (Promega^®^, Madison, WI, USA). Primers were designed in order to have the restriction sites that allow the correct insertion into the shuttle vector pVVAP (V. Visa and M. McNeil; unpublished). All oligonucleotides used were purchased from Thermo Scientific (Waltham, MA, USA) and are listed in Table 1. DNA amplification, plasmid isolation and electrophoresis were carried out using standard techniques [31]. All constructs used in this study were validated and verified by nucleotide sequencing.

### 2.4. One Step Growth and Single Burst Experiments

One-step growth curves and burst size determination assays [32] were adapted to mycobacteria and carried out in exponential growth phase cell cultures [21]. Briefly, 10^8^ *M. smegmatis* cells were suspended in 1 mL of a phage suspension at 10^8^ plaque forming units (PFU)/mL. After 50 min of adsorption at 37 °C, nonadsorbed phages were inactivated with 100 µL of 0.4% H_2_SO_4_ for 5 min followed by neutralization with 100 µL of 0.4% NaOH. The mixture was diluted 1:100 in 7H9 media and aliquots were taken at intervals of 30 min to quantify the number of phage particles [21]. The obtained results are means of three independent experiments.

A similar procedure was used for burst size determination except that 10 µL of infected cells were diluted in supplemented 7H9 in order to obtain one infected cell/mL. Then, 50 mL of the infected culture was aliquoted into 1 mL volumes and incubated for 3 h at 37 °C. Each sample was plated with 200 µL of *M. smegmatis* cells and top agar (4 mL) on 7H10 medium and incubated at 37 °C for 24 h. Phage plaques were counted, and the Poisson distribution of (P(n)) was applied to determine the burst size (BS): *P*(*n*) = (*e***^−^***^c^* × *c^n^*)/*n*! (*e* < 1), where *P*(*n*) is the probability of samples having *n* infected cells, *c* is the average number of infected cells per tube, and BS (total plaque count in the 50 plates)/(total number of infected cells) [21]. The obtained results are means of three independent experiments.

### 2.5. Determination of the Number of Phage Particles Released during the Infection Cycle

To determine the number of phage particles released into the supernatant or retained in cells, *M. smegmatis* was grown up to an OD_600_ = 0.5, infected with Ms6*wt* or Ms6Δ*lysB* at a MOI of 1 and incubated at 37 °C for 3 h. Aliquots were taken at 90 min and 180 min post adsorption and separated by centrifugation into supernatant and pellet fractions. The pellets were suspended in ice-cold phage buffer and sonicated twice for 5 s, with a 30 s interval. Each supernatant and the sonicated pellets were serially diluted using phage buffer and plated on a top agar lawn of *M. smegmatis* to determine the number of phage particles. Data represent the mean of three independent experiments.

### 2.6. Cryo-Transmission Electron Microscopy Sample Preparation, Imaging and Image Processing

To observe the lysis phenotype of Ms6*wt* or Ms6Δ*lysB*, cells were infected as described above for the one step growth experiment except that the phage input was 100-fold higher. At each time point, 200 µL aliquots were mixed with 10-nm gold nanoparticles (Sigma-Aldrich^®^, St. Louis, MO, USA). The nanoparticles were later used for image alignment in the 3D tomographic reconstruction process [33,34]. Four µL of the pre-mixed samples were applied to TEM grids that were vitrified by rapid immersion in liquid ethane using a Gatan CryoPlunger3 (Cp3) apparatus (Gatan, Pleasanton, CA, USA). Cryo-grids were transferred to a Gatan 914 high-tilt holder maintained at −178 °C. Cryo-specimens were imaged with JEOL JEM-2200FS 200-kV field emission gun transmission electron microscope (JEOL Ltd., Tokyo, Japan) equipped with an in-column Omega energy filter (slit width 20 eV), a Gatan US4000 4k × 4k CCD camera, and a Direct Electron DE-20 direct detector (Direct Electron, LP, San Diego, CA, USA). Projection images and tilt series were acquired using SerialEM software (http://bio3d.colorado.edu/SerialEM/) [35]. Single-axis tilt series were collected over an angular range of −62° to 62°, with a 2° tilt increment using the DE-20 direct detector. The total electron dose applied to the specimens did not exceed 120 e^−^/Å^2^. Tilt series images were acquired at 10,000× nominal magnification (calibrated pixel size of 0.614 nm) with −4 to −8 μm defocus applied. Tomographic reconstructions were generated with IMOD using the r-weighted back-projection algorithm [33,34].

### 2.7. Nucleotide Sequence Accession Numbers

The phage genome sequences provided in Figure S1 were obtained from GeneBank. The accession numbers are AF022214 for D29, DQ398047 for PBI1; AF319619 for Ms6, DQ398042 for Halo, AY129338 for Omega, GU580941 for ReqiPepy6, GU580940 for ReqiDocB7, KU963246 for SoilAssassin, KX557278 for Ghobes and KR053196 for TPA4.

## 3. Results

### 3.1. Ms6 LysB Deletion Decreases Viral Progeny Release

**Figure 2:**
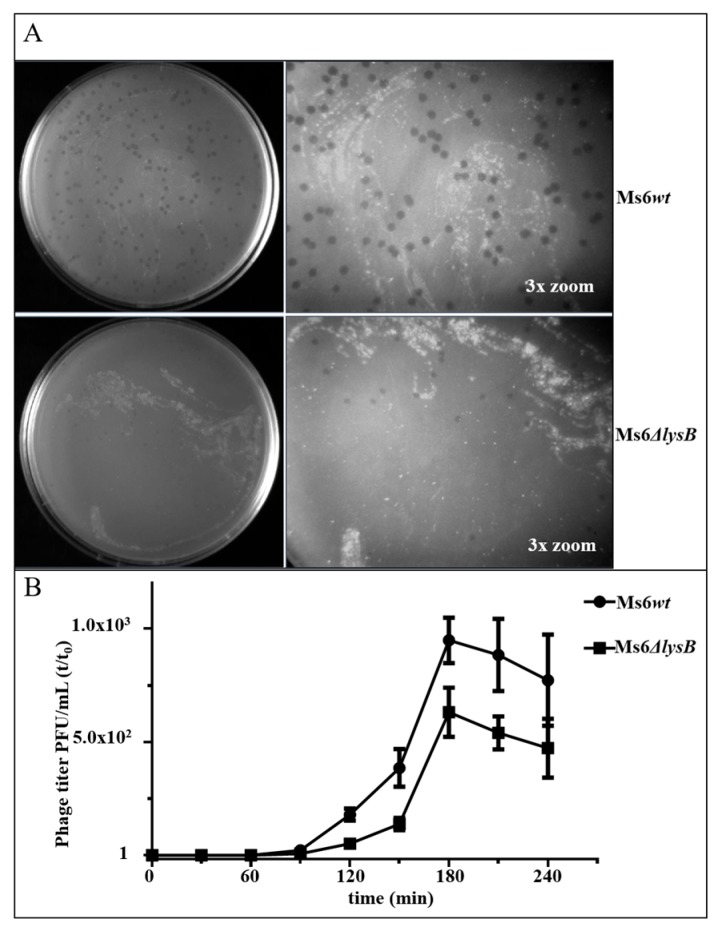
(**A**) Phage plaques formed by Ms6 (top) or Ms6Δ*lysB* (bottom) on a lawn of *M. smegmatis*. The plaques formed by Ms6Δ*lysB* phage are smaller than the ones formed by the wild-type Ms6; (**B**) one-step growth curves of Ms6*wt* (circles) or Ms6Δ*lysB* (squares) on *M. smegmatis* mc^2^155 show a lower number of plaque-forming units (PFU) released from Ms6Δ*lysB* infection. Both curves show similar progression up to 90 min post-adsorption showing no differences in the timing of lysis. T0 marks the end of the adsorption and start of the one-step experiment. The PFU/mL at t = 0 was used to normalize PFU/mL of each time point. For each time point, the mean ± SD of four independent assays is indicated.

To understand how Ms6 LysB contributes to phage-induced lysis, we took advantage of the Bacteriophage Recombineering of Electroporated DNA (BRED) strategy [30] and constructed an Ms6 derivative mutant lacking gene *lysB*. The Ms6Δ*lysB* was able to form plaques on *M. smegmatis* at equivalent efficiencies to that of the wild-type (*wt*); however, a reduction in plaque size produced by the mutant was observed (Figure 2A). In a complementation assay, where LysB production was provided from plasmid pAG1, the wild-type phenotype was restored, indicating that plaque size reduction is a consequence of LysB absence.

To test whether this phenotype results from changes in the phage growth parameters, one-step growth and single-burst experiments were performed. *M. smegmatis* cells were infected with Ms6*wt* or Ms6Δ*lysB* at a multiplicity of infection (MOI) of one. The one-step growth curves (Figure 2B) obtained for Ms6*wt* and Ms6Δ*lysB* show that the latent period is similar and that LysB has no effect on the lysis timing; however, the number of infective particles released after Ms6Δ*lysB* infection was lower than in an Ms6*wt* infection. Single-burst experiments performed to compare the viable progeny released from single cells show that a Ms6*wt* infection released an average of 147 ± 27 viable phages per bacterium, while Ms6Δ*lysB* yielded a reduced burst size of approximately 53 ± 14, where the ± values indicate the mean SD of three independent experiments. Again, when LysB was provided in *trans*, the *wt* burst size was restored. These results show that, although Ms6Δ*lysB* can accomplish lysis of the host cell, the overall process seems to be less efficient.

### 3.2. Ms6 Is Trapped in Cell Debris in Absence of LysB

**Figure 3:**
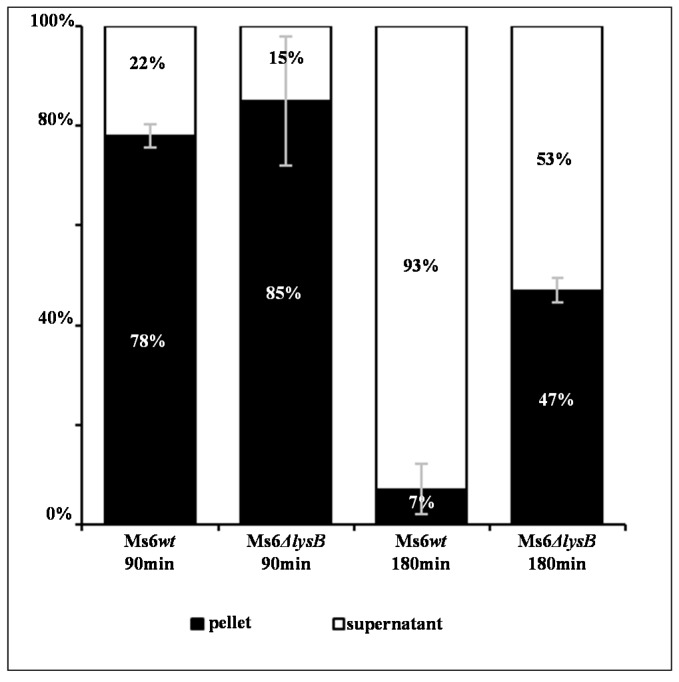
Distribution of phage particles in the supernatant and pellet of *M. smegmatis* infected with Ms6*wt* or Ms6Δ*lysB*. Ms6 is trapped in cell debris in absence of LysB. At the indicated time points, the distribution of phage particles in the pellet and in the supernatant was determined as a percentage of the total amount of PFU counted in both fractions. The values indicate the mean ± SD of three independent experiments.

Taking into consideration the observed lysis defect and that: (i) LysB is produced at a late stage of the infection cycle as other lysis proteins, (ii) this protein is a lipolytic enzyme that cleaves the linkage of the mycobacterial OM to the mAGP complex; we hypothesize that the reduced burst size results from a release defect and not from a reduction in the number of synthesized phage particles. To address this question, we performed a time course infection assay with either Ms6*wt* or Ms6Δ*lysB* and at each time point the cell pellet was separated from the supernatant and the number of phage particles in each fraction was determined. As observed in Figure 3, at 90 min post-adsorption the majority of the phage particles are not yet released and the number of PFU in the supernatant is similar for both phage infections. However, at 180 min post-adsorption, for the *wt* phage infection over 90% of phage particles are free in the supernant and only 7% are in the pelleted fraction, while for the Ms6Δ*lysB* infection, a remarkable 47% of total phage progeny is retained in the pellet. These results confirm that the reduced number of phage particles obtained for the mutant phage, in the single burst experiment, results from a deficient cell lysis, where part of the newly synthesized virions are trapped in incompletely lysed cells.

### 3.3. Cryo-EM Shows Incomplete Cell Lysis in Absence of Ms6 LysB

To prove that the unreleased phage particles remained trapped in incompletely lysed cells, we used cryo-electron microscopy (cryo-EM) and cryo-electron tomography (cryo-ET). This method allows us to visualize the host cell lysis and the viral progeny in their native environment and to examine the lysis behavior of *M. smegmatis* infected with Ms6*wt* or Ms6Δ*lysB*. From each infected *M. smegmatis* culture, with either Ms6*wt* or Ms6Δ*lysB*, aliquots were plunge frozen on copper grids for cryo-EM assessment.

**Figure 4:**
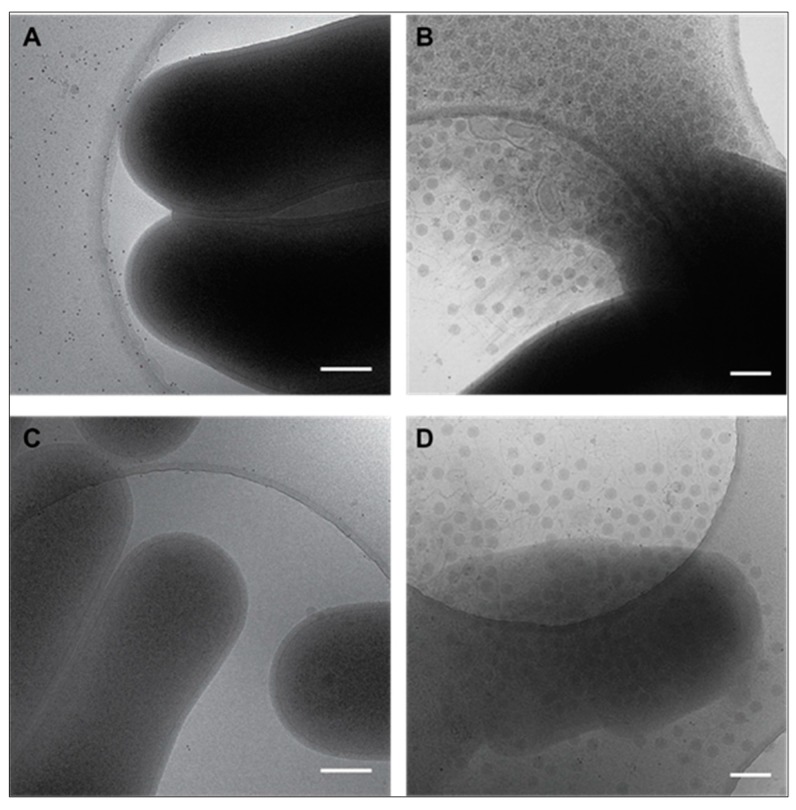
Cryo-EM images of *M. smegmatis* infected with Ms6*wt* or Ms6Δ*lysB*. At 90 min post-adsorption, cells infected with Ms6*wt* (**A**) or Ms6Δ*lysB* (**C**) are still intact and no difference is observed. At 150 min post-adsorption, the abrupt burst of a cell infected with Ms6*wt* is clear (**B**) while cells infected with Ms6Δ*lysB* (**D**) do not lyse abruptly and deformations in the cell envelope are clearly visible. Scale bar (200 nm).

Figure 4 shows collected images of infected cells at 90 and 150 min post-adsorption. At 90 min, no lysis is yet observed (Figure 4A,C). At 150 min post-adsorption, cells infected with Ms6*wt* burst and release almost all the phages (Figure 4B), while cells infected with Ms6Δ*lysB* show incomplete lysis and many phages are not released (Figure 4D). Incompletely lysed cells are still captured up to 240 min post-adsorption with the mutant phage, while for the wild-type infection only free phage particles and cell debris are observed.

**Figure 5:**
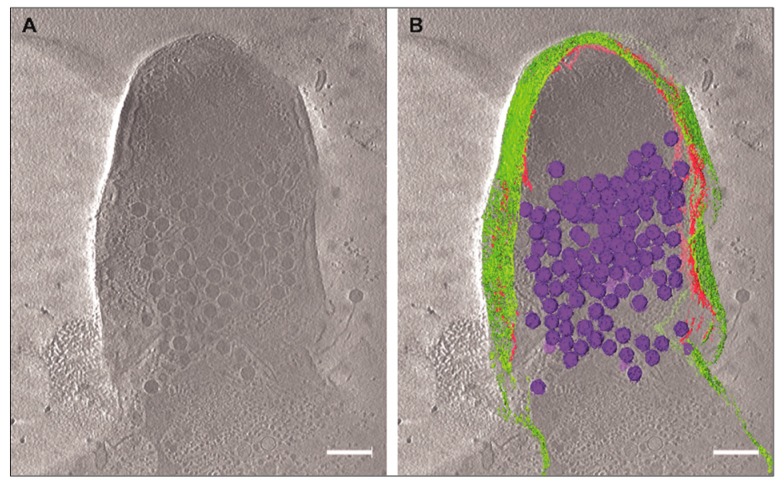
Cryo-electron tomography of *M. smegmatis* infected with Ms6Δ*lysB* at 150 min post-adsorption. (**A**) Slice through the tomogram of an infected cell; (**B**) segmented volume of the phage capsids (purple), cell membrane and PG (magenta) and the outer membrane (green). Scale bar (200 nm).

Cryo-ET data collection was performed on Ms6Δ*lysB* infected cells. In Figure 5A, a central slice through the 3D tomogram shows phages inside the incompletely lysed cell and what appears to be lesions throughout the cell envelope. To facilitate the visualization of the phages and to demonstrate they are inside the cell, segmentations of several 3D tomographic volumes was performed. With this method, it is possible to render the structures or regions of interest in the tomogram (Figure 5B). It is clear that many phages are inside the incompletely lysed cell despite the evident deformation of the cell envelope. It is also clear that most of the CM and PG are absent, while the OM still remains as a veil surrounding and holding some of the cell content.

## 4. Discussion

It is well known that lysis of the bacterial host is the last event of dsDNA phage lytic cycle, so that the new synthesized phage particles may be released into the environment and infect new available hosts. Compromising the bacterial cell barriers is a sine qua non condition to achieve this final step. Although the role of holins and endolysins has long been well defined, targeting the CM and the CW respectively, the importance of spanins in lysis has only recently been established [9]. The best characterized spanin is that of phage λ, which is composed of two subunits, the Rz and Rz1 proteins, that, once localized to the inner and outer membranes, respectively interact by the C-termini of their periplasmic domains to form a complex that spans the entire periplasm [7,10]. For many years, *Rz/Rz1* were considered auxiliary genes, because under laboratory conditions λ lysis could be achieved in the absence of these genes, unless the OM was artificially stabilized by the presence of millimolar concentrations of Ca^2+^ [36]. Recently, Berry et al. [7,9] have demonstrated that, in nature, in absence of stabilizing cations, these proteins are required for λ lysis, as induction of λ lysogens in the absence of a spanin function results in lysis failure. The infection cycle terminates leading to spherical cells where the CM and PG have been disrupted, but the OM remains intact, indicating that the latter is an important barrier to lysis. In a λ lytic cycle, the Rz-Rz1 complexes accumulate in the envelope during the morphogenesis phase. It was suggested that, following PG disruption by the endolysins, the spanins function by fusing the inner and outer membrane, this results in outer membrane disruption and consequently cell lysis [7,9].

This spherical phenotype in the absence of spanins has also been observed in infections with phages P2 [37] and PRD1 [11]. The presence of *Rz/Rz1* equivalents in the lysis cassette, of nearly all bacteriophages that infect Gram-negative hosts, strengthens the idea that to accomplish lysis, in addition to compromising the CM and the CW through the action of holins and endolysins, these phages also need to disrupt the OM [7,10].

In this work, we show that mycobacteriophage Ms6, a phage that infects the mycobacterial species, *M. smegmatis,* in addition to the holin and endolysin functions also requires an additional lysis protein to overcome the last cell barrier. We provide evidence that Ms6 LysB parallels the function of spanins.

Mycobacteria, which are members of the Corynebacteriales order, are bacteria that, despite being classified as Gram-positive, share a complex cell envelope. In addition to a CM and a CW, they also contain an OM, which is an asymmetrical bilayer where the inner leaflet, composed of long chain mycolic acids, is linked to the CW through an ester bond to AG. This peculiar OM confers to mycobacteria their characteristic impermeability and resistance to therapeutic agents, and as so, is also predicted to be a barrier to mycobacteriophage-induced lysis [13,14,15,16,17]. We have previously shown that Ms6 LysB is a lipolytic enzyme that cuts the linkage between AG and MA on the mycolyl-arabinogalactan-peptidoglycan complex [24,25].

We have observed that, in contrast with Ms6 LysA [23] and in general with phage endolysins, the Ms6 LysB, under our laboratory conditions, is not essential for the phage life cycle, since the Ms6 derivative mutant lacking gene *lysB* is viable and capable of forming plaques in *M. smegmatis*. We have observed, however, a reduction in the plaque size. In a one-step assay, we could demonstrate that in absence of LysB there is a defective phage release at the end of an infection cycle. Indeed, in the single burst experiment a reduction of 64% in the number of free phage particles per bacterium was observed. This is in agreement with the reduced plaque size of Ms6Δ*lysB*, a phenotype that was reverted to the wild-type when LysB was provided in trans. Since Ms6 LysB is produced during late gene expression, from gene *lysB*, which is part of the lysis cassette, a role in host lysis is obviously expected.

A reduced phage release, together with a reduced plaque size was also reported for the LysB of mycobacteriophage Giles; however, and in contrast to phage Ms6 where absence of LysB did not affect the timing of lysis, the authors observed that lysis induced by GilesΔ*lysB* was delayed in 30 min when compared to the Giles*wt* [38]. It is not clear so far how the absence of Giles LysB affects the timing of lysis.

The observation that, at the end of a Ms6Δ*lysB* infection, 47% of the phages are recovered from the cell pellet against only 7% in a Ms6*wt* infection, indicates that the reduced burst size results from a deficient phage release and not from a reduction in the number of new synthesized phage particles. In the absence of LysB, phage particles are trapped in incompletely lysed cells. Cryo-EM and cryo-ET of infections in the absence of LysB clearly show unreleased phage particles inside cells infected with Ms6Δ*lysB*, while at the same time point (150 min post-adsorption) in a Ms6*wt* infection the cell completely bursts. The 3D tomogram (Movie S1) also shows deformations of the cell, indicating that the OM still holds part of the cell content even after disruption of the CM and PG (Figure 5B), following holin and endolysin action.

Our results support the notion that the role of Ms6 LysB in lysis equates to that of spanins, however with different modes of action, since the structure of mycobacteria OM is completely different from that of Gram-negative bacteria. While spanins function either as a complex (λ Rz-Rz1) or as a single protein (T1 Gp11) by fusing the CM and OM [39], Ms6 LysB protein functions as an enzyme that detaches the OM from the CW by cleaving the bond that links these two structures. As a lipolytic enzyme, Ms6 LysB also acts as an esterase on other lipids containing mycolic acids, such as the trehalose dimycolate (TDM) [25], a glycolipid with an important role in *M. tuberculosis* pathogenesis [40]. However, it is unknown if cleavage of these lipids contributes to lysis.

The fact that the vast majority of mycobacteriophages sequenced so far encode Ms6 LysB homologous proteins suggests that they have an important role in nature. This is also true for other phages that infect members of the mycolata group, a bacterial group that also contain a layer of mycolic acid-containing lipids in their envelope. Examples are the *Rhodococcus equi* phages ReqiDocB7, ReqiPepy6 and ReqiPoco6, which encode Ms6 LysB homologues [12,41]. A huge number of genome sequences from phages infecting the same bacterial group is available at The Actinobacteriophage Database (http://phagesdb.org/), and here we can also find genes from several phages annotated as coding for Lysin B as exemplified by *gp24* or *gp41* from phages SoilAssassin and Ghobes, respectively, both infecting *Gordonae terrae* [42]. In other cases, although no LysB annotation exists, we could identify the GXSXG motif common to lipolytic enzymes in the deduced amino acid sequence of several genes, such as *gp54* from the TPA4 phage, a lytic phage that infects Tsukamurella species (Figure S1).

Collectively our results lead to the suggestion that mycobacteriophage-induced lysis is also a three-step process where holins subvert the cytoplasmic membrane followed by endolysins targeting the cell wall and LysB proteins disrupting the last barrier to mycobacteriophage release, the outer membrane.

Our present knowledge of the mechanism of bacteriophage lysis suggests that the complexity of phage lytic cassettes depends on their hosts. Hosts with a simpler envelope, like Gram-positive bacteria, require the phage to possess a simple lytic cassette, with genes encoding proteins targeting the CM and the PG. For bacteria with a more complex envelope that also contain an OM, degradation of the cell wall is necessary but not sufficient for lysis and phages need to produce specific proteins to overcome this barrier. Thus, phages that infect Gram-negative hosts or mycobacteria, in addition to holins and endolysins, synthesize spanins or lipolytic enzymes, respectively (Figure 1).

## Figures and Tables

**Table 1:** Bacterial strains, phages, plasmids and oligonucleotides used in this study.

Name	Description	Source or Reference
Bacteria		
*Mycobacterium smegmatis* mc^2^155	High-transformation-efficiency mutant of *M. smegmatis* ATCC 607	[26]
Bacteriophages		
Ms6*wt*	Temperate bacteriophage from *M. smegmatis*	[27]
Ms6Δ*lysB*	996 bp in-frame deletion of the Ms6 *lysB* gene	This study
Plasmids		
pJV53 pAG1	Derivative of pLAM12 with Che9c *60* and *61* under control of the acetamidase promoter; Kan^r^ *lysB* gene cloned into pVVAP	[28] This study
pVVAP	Mycobacteria shuttle vector carrying the acetamidase promoter; Kan^r^	[29]
Oligonucleotides	Sequence 5’-3’ ^a^	
PrΔ*lysB*	CTCGGCGGAAAAACCCTCCTCGTGGACGCGGTAGCAGAACTGTTGGGCCACTGATAGGAGGCACCCATGCTGACACGTTCATTCTGGATCGACGCCGCCGAGCG	Ms6Δ*lysB*
PrExtΔ*lysB*Fw	CGAGATCCTGCGGCAACTGCGCGGATACAACCTCACTGGCTGGCCGCAGCTCGGCGGAAAAACCCTCGTGGACG	Extend Pr∆*lysB*
PrExtΔ*lysB*Rv	CCCCGGCGCCGAGGGTGGCGATCGCGGTTTGGGCGAATGTGCGTATGGCACGCTCGGCGGCGTCGATCCAGAATG	Extend Pr∆*lysB*
Pr*lysB*Fw	GCGGATCCATGAGCAGAACTGTTGGGCC	Includes BamHI site to clone in pVVAP
Pr*lysB*Rv	GGAAGCTTTGTGCGTAGGTAGTCGATG	Includes HindIII site to clone in pVVAP
DADA Δ*lysB* PCRFw	GCGCTAGCAGAACTGTTGGGCCACTGATAG	Ms6Δ*lysB*
DADA Ms6-PCRRv	CGTCTCGTACTGCACGTACCGGTTCTTC	Ms6Δ*lysB*

^a^ Restriction sites are underlined.

## Supplementary Material

The following are available online at www.mdpi.com/1999-4915/9/11/343/s1.
