# Supplementary material for: The Ms6 Mycolyl-Arabinogalactan Esterase LysB is Essential for an Efficient Mycobacteriophage-Induced Lysis
Source: Viruses. 2017 Nov 17;9(11):343. doi: 10.3390/v9110343 (PMC5707550; doi:10.3390/v9110343)
Supplement: Supplementary file 1 [file viruses-09-00343-s001.zip › suppl/Figure S1.pdf]

Figure S1

A

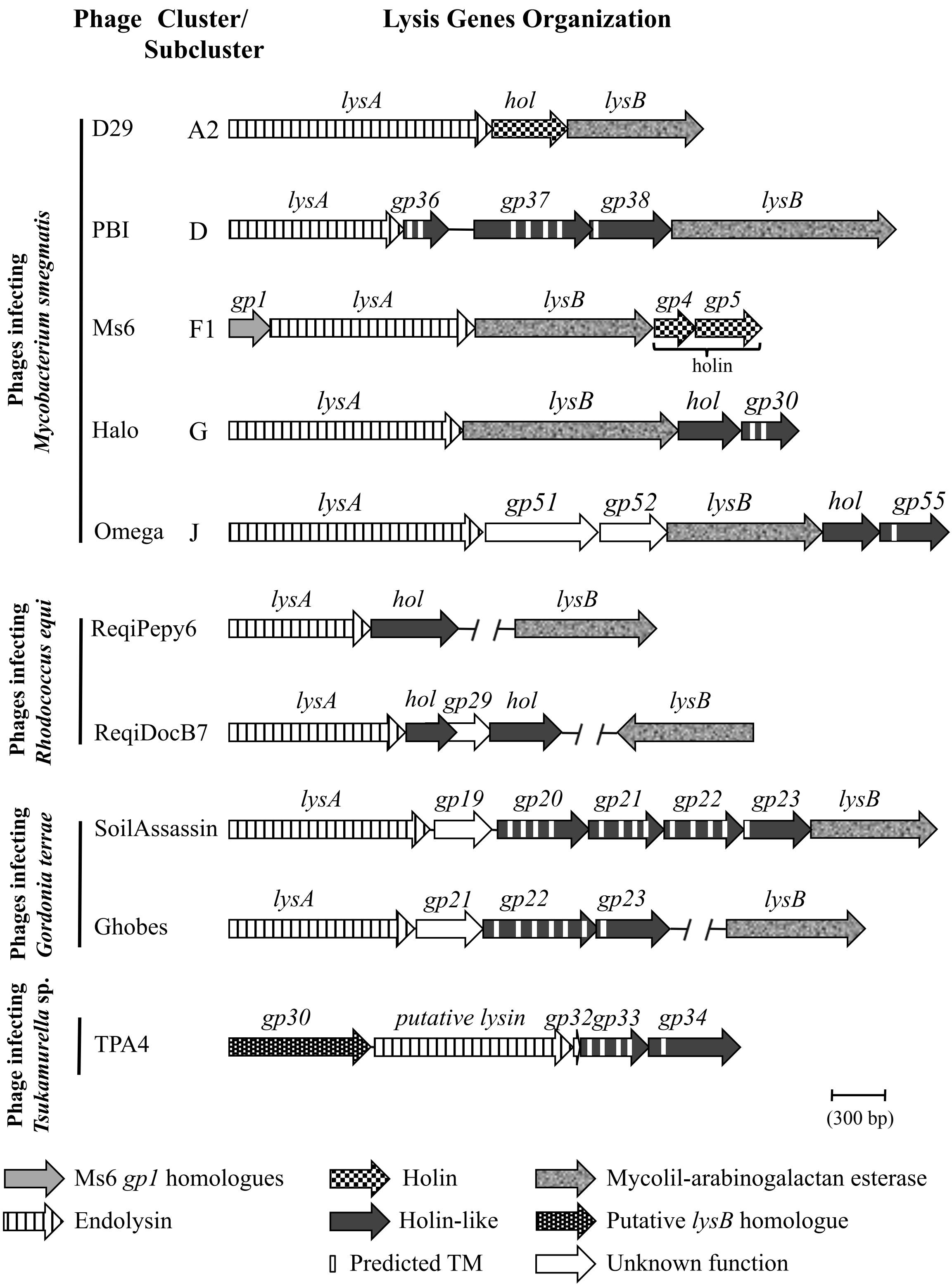
Phage Cluster/ Subcluster
Lysis Genes Organization
Phages infecting *Mycobacterium smegmatis*
D29 A2
lysA hol lysB
PBI D
lysA gp36 gp37 gp38 lysB
Ms6 F1
gp1 lysA lysB gp4 gp5
holin
Halo G
lysA lysB hol gp30
Omega J
lysA gp51 gp52 lysB hol gp55
Phages infecting *Rhodococcus equi*
ReqiPepy6
lysA hol lysB
ReqiDocB7
lysA hol gp29 hol lysB
Phages infecting *Gordonia terrae*
SoilAssassin
lysA gp19 gp20 gp21 gp22 gp23 lysB
Ghobes
lysA gp21 gp22 gp23 lysB
Phage infecting *Tsukamurella* sp.
TPA4
gp30 putative lysin gp32 gp33 gp34
(300 bp)
Ms6 *gp1* homologues
Endolysin
Holin
Holin-like
Predicted TM
Mycolil-arabinogalactan esterase
Putative *lysB* homologue
Unknown function

B

```
D29            70  ...ADPYADFAMAGYSQGAIVVGQVLKH...  95
PBI1          168  ...KDPDVEFWFSGYSQKADGLEDALEI... 193
Ms6           156  ...RIETHGTALAGYSQGAVVLSELWMN... 181
Halo          175  ...VNPKIRVVIGGYSAGAIAAAMF-RA... 199
Omega          85  ...RTTPGKIVLSAYSQSAVAFAYVWRD... 110
ReqiPepy6      76  ...SNPGKQFILLAYSGGNRVIHEW-ME... 100
ReqiDocB7      53  ...-FTRQAIFVAGYSQGGTVVTKAIKH...  77
SoilAssassin 71  ...ERSQFDVVVAGYSQGAAVAVHY-AL...  95
Ghobes         77  ...AHPDDRFVVLGYSLGALVGTKF-LE... 101
TPA4           73  ...AAARGRFVGLGYSQGGSLLTEWLNR...  98
```

Figure S1 Comparison of *lysB* genes from phages infecting members of the Mycolata group**. (A)**Illustrated are representatives of mycobacteriophages and phages infecting *Rhodococcus equi, Gordoniae terrae* and *Tsukamurella* spp.. The genes marked with a white segment indicate genes not previously assigned as holins, but having predicted transmembrane segments. **(B).** Alignment of Ms6 LysB and putative LysB protein homologues. The conserved pentapeptide (G/A-X-S-X-G) is highlighted on a grey background. Numbers refer to the amino acid positions.
