# Supplementary material for: The Ms6 Mycolyl-Arabinogalactan Esterase LysB is Essential for an Efficient Mycobacteriophage-Induced Lysis
Source: Viruses. 2017 Nov 17;9(11):343. doi: 10.3390/v9110343 (PMC5707550; doi:10.3390/v9110343)
Supplement: Supplementary file 1 [file viruses-09-00343-s001.zip › suppl/Movie SI legend.pdf]

Movie SI. Sequence of cryo-electron tomography slices of *M. smegmatis* infected with Ms6Δ*lysB* at 150 min post-adsorption. The middle section slices, show deformations of the cell envelope and many icosahedral phage capsids (Ms6Δ*lysB*), indicating that the OM still holds part of the cell content even after disruption of the CM and PG. Scale bar 200 nm.
